# The Italian interregional performance evaluation system

**DOI:** 10.1007/s43999-022-00010-6

**Published:** 2022-09-16

**Authors:** Federico Vola, Vera Benedetto, Milena Vainieri, Sabina Nuti

**Affiliations:** Management and Healthcare Laboratory, Institute of Management and Department EMbeDS, Scuola Superiore Sant’Anna, Via San Zeno, 2, Pisa, PI 56127 Italy

**Keywords:** Health System Performance Assessment, Performance evaluation systems, Resilient health systems

## Abstract

This contribution describes the Interregional Performance Evaluation System (IRPES) designed by MeS Lab of Sant’Anna School of Advanced Studies (Pisa—Italy), by highlighting its main characteristics and its evolution.

**Context:** Since its creation in 2008, the IRPES has been developed by researchers from different backgrounds, with the involvement of practitioners and managers, so as to offer support to local, regional and national healthcare management. The IRPES is currently adopted by 12 Italian regions, which share a common performance evaluation system to assess the respective regional healthcare systems’ performance.

**Structure: **The IRPES is currently composed of about 400 indicators, which monitor different dimensions of the health systems. About half of them are benchmarked against shared standards, to assess the healthcare systems’ performance. Indicators are grouped into around thirty composite indicators.

**Data visualization:** MeS Lab designed innovative tools to deliver a clear representation of the performance of health systems, which in turn facilitate policymakers to gain a dynamic understanding of weaknesses and strengths of the systems they manage. In particular, the more traditional representation tools (such as maps or histograms) are accompanied by new visualisation instruments, such as “the dartboard” and “the stave”.

**Covid-19: **With the outburst of COVID-19 pandemic emergency, assessing the resilience of healthcare systems has become the new challenge posed to the IRPES. Thus, 63 novel resilience indicators tailored for the pandemic were introduced, starting from the second semester of 2020. Continuous monitoring of the performance of health systems was equally implemented, which has been essential to policymakers during such a difficult time.

## Introduction

### The Italian national health service

With a population of almost 59 million (in 2022), Italy is the sixth most populous country in Europe. The country is made up of 20 regions, which are extremely varied, differing in size, population and levels of economic development. Since the early 1990s, considerable powers, particularly in healthcare financing and delivery, have been devolved to this level of government. Italy’s healthcare system is therefore a regionally organised National Health Service (Servizio Sanitario Nazionale, SSN) that provides universal coverage largely free of charge at the point of delivery. At national level, the Ministry of Health (supported by several specialised agencies) sets the fundamental principles and goals of the health system, determines the core benefit package of health services guaranteed across the country, and allocates national funds to the regions. The regions are responsible for organizing and delivering health care: they have progressively developed significantly different organizational models associated to heterogeneous performances in providing healthcare services [1–6].

At local level, geographically based local health authorities (Aziende Sanitarie Locali) deliver public health, community health services and primary care directly, and secondary and specialist care directly or through public hospitals or accredited private providers (*OASI *[7]* | Cergas*, n.d.). In almost all demographic and health indicators, there are marked regional differences for both men and women, reflecting the economic and social imbalance between the north and south of the country [8, 9].

### Health system performance assessment in Italy

Evaluating the performance of health systems is notoriously a complex task. Identifying the most efficient way to assess quality and performance in delivery of care, and developing indicators that can be a contribution for the main stakeholders involved in the decision-making process is a demanding and laborious process [10].

Three main Health System Performance Assessment (HSPA) tools are currently implemented in Italy, in order to assess the national and regional health system performance [10]:

1. the *National outcome evaluation programme* (PNE) has been developed by the National agency supporting regional health systems (AGENAS), to monitor healthcare outcomes at the regional and local (hospitals and municipalities) level [11, 12]*—Programma Nazionale Esiti*, n.d.),
2. the Ministry of health has developed its HSPA tool since 2005 [13]; the tool has been substantially renewed starting from 2020 (Nuovo Sistema di Garanzia—NSG): it is now composed by 88 indicators, computed with regional granularity, in order to mainly assess structure, output and process indicators [14]
3. the Italian Interregional Performance Evaluation System (IRPES).

### The Italian interregional performance evaluation system

This article describes the Italian Interregional Performance Evaluation System (IRPES), a tool designed by the Management and Health Lab (Laboratorio Management e Sanità – MeS Lab) of Sant’Anna School of Advanced Studies (SSSA), Pisa (Italy).

The Performance Evaluation System (PES) and the Interregional Performance Evaluation System (IRPES), respectively implemented in 2004 and 2008 by the MeS Lab of Sant’Anna School of Advanced Studies, represent a voluntary based governance tool to support healthcare managers and policy makers at regional and local level [15–18]. Specifically, the PES instrument was designed and developed by the MeS Lab after a request by the Tuscany region,by 2006, the system was adopted by all healthcare Tuscan organisations. Two years later, an interregional collaboration was instituted (initially including Tuscany, Liguria, Umbria and Piedmont), in order to share an evaluation system to assess respective healthcare systems’ performance. The IRPES was created as a common set of indicators and a shared methodology to assess them.

Adhering to the IRPES requires both political commitment – in order to endorse full responsibility for public disclosure of healthcare performance and to effectively link performance measurement with performance management tools – and technical capacity, to be able to rapidly compute hundreds of complex indicators in a limited time. These two compelling requirements explain why some Regions chose to interrupt data sharing after testing the tool.

Basilicata, Friuli Venezia Giulia, Liguria, Lombardy, Marche, Piedmont, Puglia, Tuscany, Umbria, Veneto and the Autonomous Provinces of Bolzano and Trento are part of the system in 2020. Table 1 illustrates the regional adhesion to the IRPES from its constitution onwards.

**Table 1:**
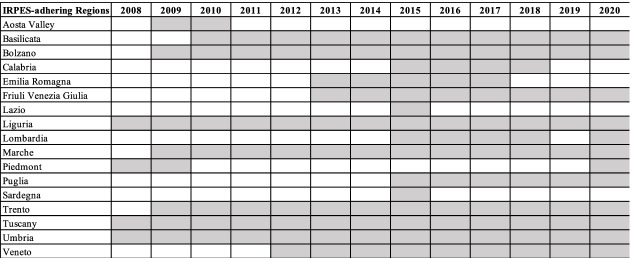
Regional adhesion to the Interregional Performance Evaluation System (IRPES)

The IRPES is designed to support regions in measuring, comparing, and portraying the degree of quality, efficiency, appropriateness, continuity of care, and patient and staff satisfaction achieved by the respective healthcare systems [19]. The comparison can be performed not only at a regional level, but also at an intra-regional one, between the health structures of each Region. Moreover, in order to deepen the intra-regional or intra-provincial assessment, regions can decide to take broad geographical areas, districts or health authorities as the unit of analysis. Starting from 2016, the MeS Lab has been publishing a report specifically tailored for university hospitals as well (Azienda Ospedaliera Universitaria – AOU) [15, 16, 18, 20–24]. Based on the benchmarking process, the regional network combines a longitudinal trend with a cross-sectional perspective. It offers key information to the regions in order to identify goals and set suitable targets while taking benchmarking results into account. Moreover, having the same performance evaluation system, the regions can identify, assess, investigate and spread best practices [25].

A process of interregional sharing has led to the selection of about 400 indicators, aimed at describing and comparing, through a benchmarking process, the different dimensions of the health systems’ performance, namely:

- The state of health of the population
- The ability to pursue regional strategies in the time and manner indicated
- The evaluation of economic-financial dynamics and operational efficiency
- The evaluation of the experience and the satisfaction of patients and of staff
- The emergency-urgency area
- Prevention
- The governance, appropriateness and quality of health services
- Pharmaceuticals.

## IRPES structure and main characteristics

This section aims at describing the structure of the IRPES. Nine main characteristics define its architecture and most of them are shared by the other above-mentioned Italian HSPA tools:

- Voluntary: One of the main features of the Interregional Performance Evaluation System regards the choice for regions to enter or to exit the network. This dimension is an important proxy of willingness of improving performances—also by possibly innovating organisational regional structures. Furthermore, the voluntary adhesion to collaborate with the research is the result of a conscious choice of the adhering regions that want, through this governance instrument, to guarantee transparency of results on the one hand, and scientific rigor on the other. Moreover, by entrusting a third and public subject, the Sant’Anna School of Advanced Studies of Pisa, the aim is ensuring the correctness of the calculation and the overcoming of self-reference.
- Public disclosure and transparency: A public report containing the results of the regions [22] and of teaching hospitals [26] is redacted annually, allowing all stakeholders, including citizens, to access it. The process of comparison between the institutions of the system—at the regional and national level, but also internationally—on the numbers, choices and results, in a transparent and public way, is not only the manner in which the public system can and must be accountable to citizens for its actions, but it also represents the essential tool for identifying areas of weakness and responsibly addressing them. Regional representatives meet on a regular basis to analyse the results of the evaluation system, identify best practices, and compare the success of various regional policies. The regular reporting of performance comparison that the IRPES provides may result in some form of regional competition. In line with the concept of transparency, since the establishment of the IRPES, a website is accessible to all, so as to offer the opportunity to everyone to be informed on the performance of health systems (https://performance.santannapisa.it/).
  The first two characteristics mentioned above have emerged in all their relevance during the COVID-19 pandemic. As a matter of fact, the systematic comparison between institutions and the timely and transparent return of results have been the preconditions to avoid self-referentiality and identify both errors and best practices and then correct the former and spread the latter.
- Evidence Based: Considering that all indicators of the evaluation system are computed by regions, the informative richness of the evaluation system is the result of the adoption of a wide range of data sources originating from a broad spectrum of administrative flows available at the national level: Hospital Discharge Records (Scheda di Dimissione Ospedaliera – SDO), balance sheets, the flow of Outpatient Services and Emergency Rooms, Birth Certificates, Pharmaceutical Flows and Home and Residential Care streams. Finally, the IRPES regularly performs ad hoc surveys to shed light on areas of interest (such as organisational climate), and specific analyses, such as assessing the research activities of university hospitals [27]. The first months of outbreak of the coronavirus pandemic have seen the growth of trust in the Italian National Health Service, but at the same time they have also offered the diffusion of different types of fake news and denialism: transparency on results, free circulation of data and information and comparison based on real world evidence are undoubtedly effective tools to stem certain drifts of public debate. Furthermore, the demanding task of matching evidence-based data with public disclosure of results, demonstrates a certain amount of willingness, preparedness and commitment of policy makers towards public accountability.
- Systematic evaluation and benchmarking: This characteristic and the feature of disclosing results publicly were identified by the literature to be the most important ones to leverage professional reputation [28–30]. Indeed, the regions of the Network have always recognised benchmarking to be essential: in a context in which collaborative and non-competitive strategies tend to be activated among the actors of the system, the systematic process of comparing performances is a fundamental tool for avoiding self-referentiality and identifying learning opportunities from best practices. Thus, the IRPES responds to the objective of providing each region with a way of measuring and representing the performance of its own local public health authorities in comparison with those of other regions, hence fostering interregional benchmarking. Moreover, the benchmarking process can also take place from an intra-regional point of view, between the health authorities (HA) of each region. To do this, PES measures results in quantitative terms and then assesses performance for 160 (evaluation indicators) of the about 400 indicators into five scores—excellent, good, sufficient, poor or very poor -, which are associated with different colours, from dark green (excellent performance), to red (poor performance). Regions define the scores by employing the same reference criteria, which are based on scientific literature, national standards, or, in the absence of these, the distribution of all considered health authorities.
- Shared design: Regular meetings with regional representatives, who include both managers and clinicians, are used to establish indicators. To be able to influence and modify behaviour, an assessment system must first secure clinicians’ approval for the norms and criteria against which their performance is assessed [31]. Moreover, indicators are developed by embracing a “managerial” attitude targeted at organisational progress [32]: the selection of each indicator is based on the informative value it may provide to managers and policymakers. Indicators are chosen not only to depict the epidemiological state of certain regions/local authorities, but also to detect best practices – at organisational level – or, on the opposite side, defective clinical processes. Each region has the responsibility for processing its own data, so as to increase the awareness and the expertise of the regional managers and their staff. During the pandemic emergency of the past years, the activity of systematic discussion with regional stakeholders has been virtuously maintained by shifting to calls and online meetings.
- Multidimensionality: In order to offer a multidimensional evaluation of a complex area such as healthcare performance, results are analysed according to different perspectives. Subsets of indicators are intended to highlight the fundamental dimensions – namely regional health strategies, efficiency and sustainability, user satisfaction, staff and communication, emergency care, governance and quality of supply, maternal and child care, chronic diseases – of healthcare performance. During the COVID-19 pandemic, the concept of multidimensionality has become of primary importance due to need of taking into account a new and important dimension, which is resilience. For this reason, the IRPES quickly integrated this dimension, without however neglecting the other ones, which still were monitored also during the emergency.
- Timeliness: Another important feature of the interregional performance evaluation system is the concept of timeliness. As a matter of fact, promptness in giving results within few months from the collection of data and continuous evaluation and improvement of indicators, offers the possibility to the main stakeholders to be continuously up to date and prepared to react based on accurate and timely results. Indeed, the publication and presentation of results takes place every year between the end of May and beginning of June, with the Report being published by the end of the year. Moreover, 2020 has posed a new challenge in terms of timeliness: due to the emergency, the need of the main stakeholders to have access to results in the most timely manner has pushed the MeS Lab towards an in-process monitoring.
- Dynamism: Evaluating the performance of a system is an intrinsically dynamic activity, which must constantly adapt to the cognitive needs of the actors in the system itself. If on a certain level the system may be static as it would otherwise become difficult to identify a yearly trend or make comparisons among different years, on the other side this assessment tool should be always embedded—hence adapted—to the context. Furthermore, 2020 has been an intense year, which required researchers to identify as quickly as possible new performance evaluation criteria suitable to measure resilience of healthcare systems during the pandemic emergency. This has led the IRPES to become even more dynamic.
- Graphical Representation: The return of the results (both on the website and in the printed reports) makes use of a wide range of graphical solutions for an immediate representation of the performance.

The indicators are grouped, through “tree” structures, into approximately thirty composite indicators, in order to facilitate the reading and interpretation of performance results. The tree structure can be described by two levels: the first one is represented by the “head indicator” (the composite indicator), which is obtained by the average - simple or weighted - of the evaluations of the sub-indicators - namely those belonging to the second level. Hence, if the reader seeks to have a summarised perspective of the performance of a certain region or health authority, the head indicators are key to offer this viewpoint. Nevertheless, if the reader wants to deepen the comprehension of a certain result related to a head indicator, the respective sub-indicators, which can be considered as the building blocks of the latter, can be drilled down and analysed.

## Data visualisation

MeS Lab is committed to adopting and developing the most innovative graphical solutions, in order to effectively convey health information to healthcare managers.

**Fig. 1:**
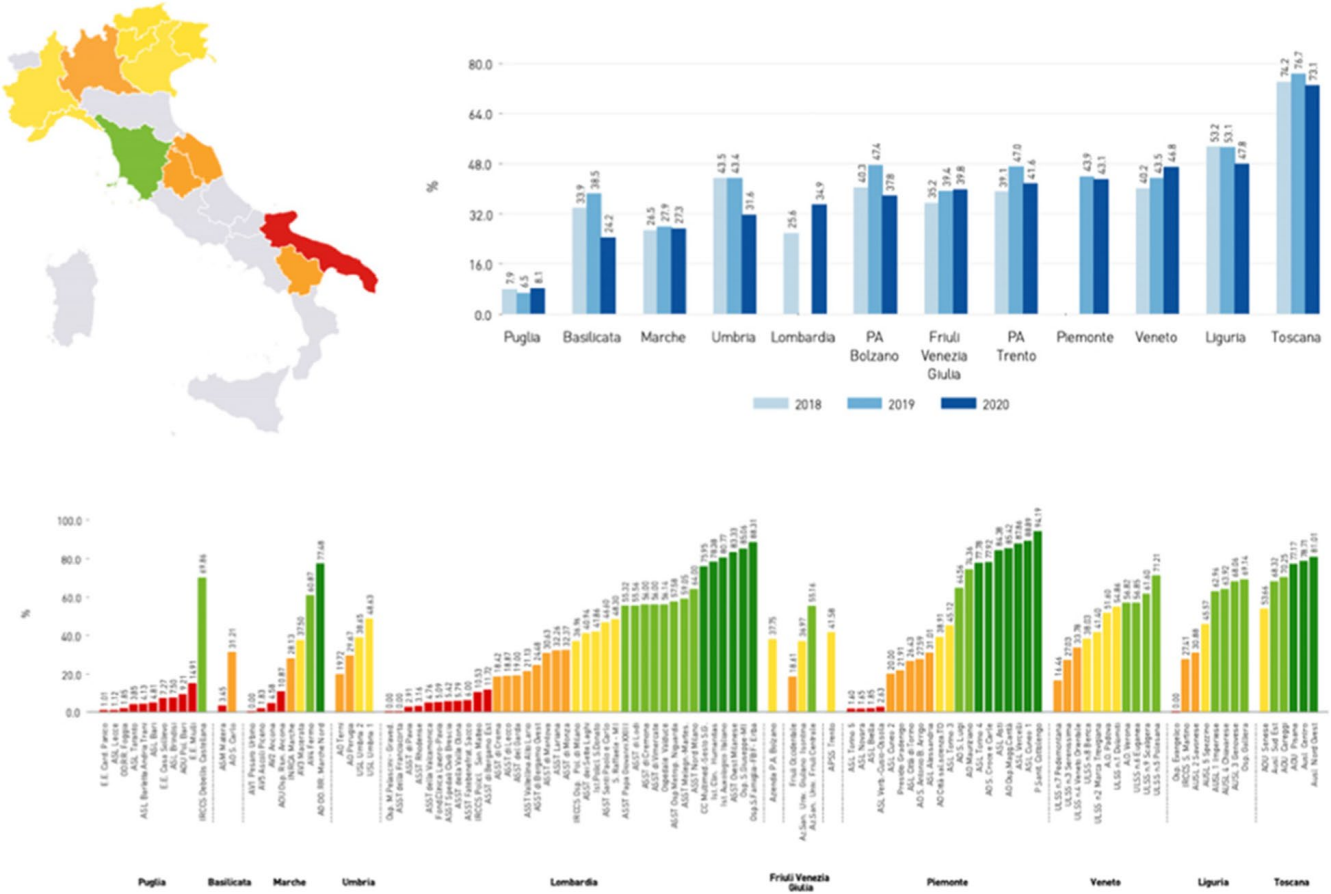
Map, trend histogram and health authorities histogram

The IRPES representation tools can be divided into two categories, namely a more classical one, opposed to a more innovative set. In the first category, it is possible to find two histograms and a map, representing each indicator: the map returns the evaluation of the reference year; the first histogram shows the regional values in comparison, with the trend compared to the years before; the second shows all the health authorities of the network in comparison, grouped by region (see Fig 1).

**Fig. 2:**
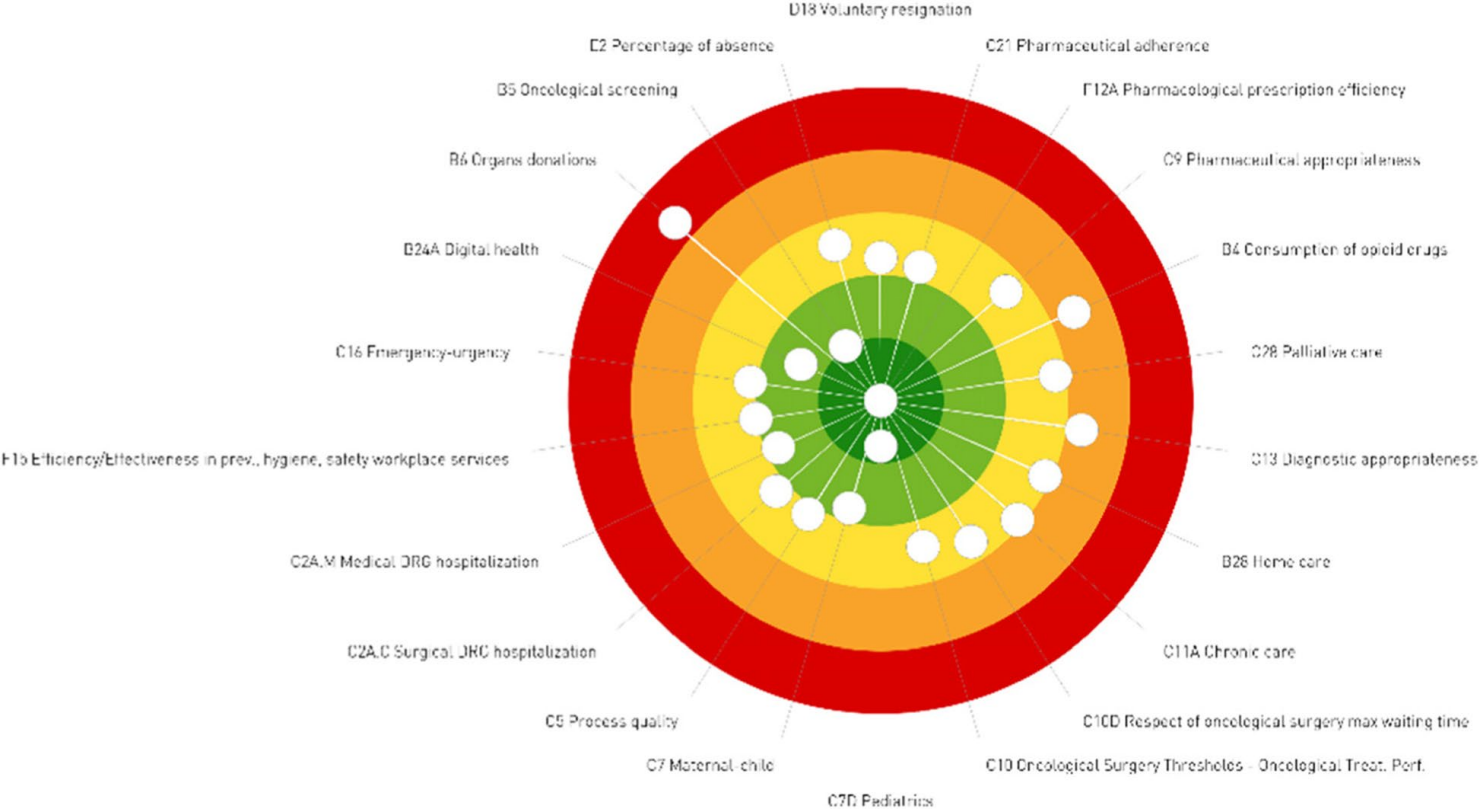
The “dartboard” of Region 1

Compared to the chart described above, the so-called “dartboard” falls in the second group, namely the category of more innovative graphical representations. With its six dimensions, it is used to display the performance of each region or HA (Fig. 2). This is divided into five (concentric) evaluation bands as well, each of which is connected with the same aforementioned distinct scores and colours. Each dot portrayed on the dartboard represents one of the composite indicators of the IRPES. When the score is high, the dot is presented in the centre (dark green), and when the score is low, it is displayed far from the centre (red). The score is annually revised and assigned for each region that voluntary participates to the IRPES based on the same reference criteria, based not only on scientific literature, but also considering national standards, or, in the absence of these, the distribution of all considered health authorities. So, if a region is performing considerably well according to literature, national standards and the median of all health authorities participating to the IRPES, their composite indicators are displayed close to the centre of the dartboard, accordingly between the green and dark green colour bands. For example, Fig. 2 illustrates the yearly performance of a certain region, whose performance can be considered good for most (composite) indicators, but needs improvement concerning organs donations.

**Fig. 3:**
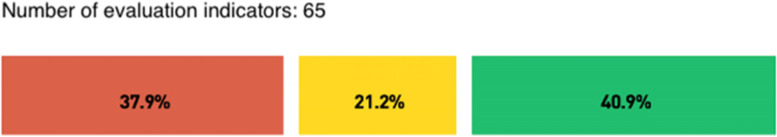
Stacked bar, that highlights the proportion of worsened, stable or improved indicators, compared to the previous year

In order to offer a representation of the dynamics of the system, each dartboard is accompanied by a stacked bar (Fig. 3) that returns—for each Region/Province/Company—the trend between the considered year and the one before, showing whether the percentage of these improved, worsened or were stable (in a range between + 1% and -1%). To offer an example, Fig. 3 indicates the ability of a certain region to have improved almost 41% of its indicators. However, almost 38% of other indicators have worsened compared to the year before.

The IRPES has incentivised professionals and other stakeholders to focalise on population value creation through the inclusion of a large set of outcome measures, also by considering the residents’ geographical area. However, until 2016, this performance evaluation tool was rooted in an “organisation-focused” approach, in which each unit and organisation's performance is monitored and reported independently. Although the data offered by this measuring method is critical for evaluating an organisation's effectiveness, focusing just on the single organisational tiles may be deceptive, given that patients' care paths frequently extend across many care settings. Emerging healthcare requirements, in reality, necessitate coordinated responses and shared responsibilities from a diverse set of providers. As a result, assessment methods must be reframed to recognise the contributions of all links in the healthcare value chain and to emphasise the shared responsibilities of the many entities involved in the care pathway. In order to overcome these limitations, the interregional performance evaluation system started merging the organisational viewpoint with the patient-centred perspective and restructuring graphical representations accordingly.

In 2016, the research team and various stakeholders realised the necessity to examine performance data at a pathway level as well [33]. The original graph—i.e. the dartboard—was combined with a new tool that represents the care pathways' performance using the metaphor of the “stave”, i.e. the set of horizontal lines and spaces used in musical notation, in order to provide an effective graphical representation by shifting the focus from single organisations' perspectives to care pathways results. Both elements of the metaphor have one thing in common: they allude to a “positive” connotation by mentioning leisure and creative pursuits. This is designed to encourage the user to have a positive viewpoint, particularly by leveraging the framing effect [34].

A selection of the initial indicators employed in the IRPES were rearranged according to the different phases that patients pass through along the paths [35, 36]. Based on their significance, four pathways have been selected, i.e. the maternal and paediatric pathway, the oncological pathway, the chronic diseases pathway and the emergency care pathway. In order to properly reflect the numerous phases that each care path is composed of, its design includes the selection of the most appropriate indicators. As the dartboard, the stave has five colour bands as well—dark-green to red. These bands are shown horizontally and designed to reflect the many stages of care paths. This approach allows users to concentrate on the strengths and weaknesses that define healthcare service delivery at various stages of the pathway [15, 16, 18, 37, 38].

**Fig. 4:**
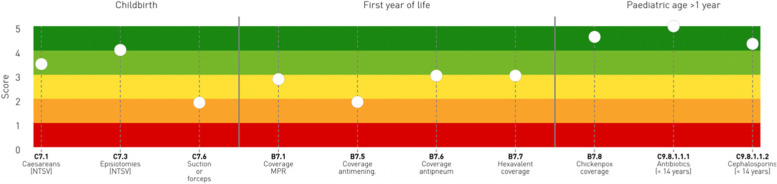
An example of the stave of Region 2 for the Maternal/Child Pathway, year 2019

Staves are employed to illustrate the performance of pathways at both regional and local levels. If on one hand, regional pathways provide information on regional performance, but do not identify the providers, on the other hand, local pathways highlight the contributions of each authority to the overall care pathway and concentrate the viewer's focus on joint value generation for each local area population. As illustrated in Fig. 4, each dot represents the performance of “Region 2” associated to each indicator along the three phases of the stave. Thus, even though in paediatric age Region 2 performs excellently, some improvements may be needed in the preceding stages of the pathways, i.e. childbirth and first year of life. Nonetheless, overall this region performs considerably well, as 50% of the dots are positioned between the green and dark green bands.

This framework has been combined with patient-related experience measures and patient-related outcomes measures – PREMs and PROMs – so as to further explore performance from patients’ perspective [36, 39–44]. These data are derived from standardised and continuous patient surveys that collect their feedback on outcomes and care experiences.

The stave achieves two purposes by adopting a pathway approach. First, by embracing the value creation model, it directs the user's focus to the patient perspective. Second, the stave emphasises the contribution that each organisation makes to the overall results of the care pathway by illustrating the performance and best practices of all organisations that serve the population of a geographical region in each pathway phase. Hence, stakeholders in the healthcare system may better grasp the importance of delivering value to their target population. Managers may be able to analyse the performance of service provision in the several stages that make up a care pathway by using this visual representation, and, as a result, assign co-responsibilities to the diverse providers engaged in each phase's service delivery.

**Fig. 5:**
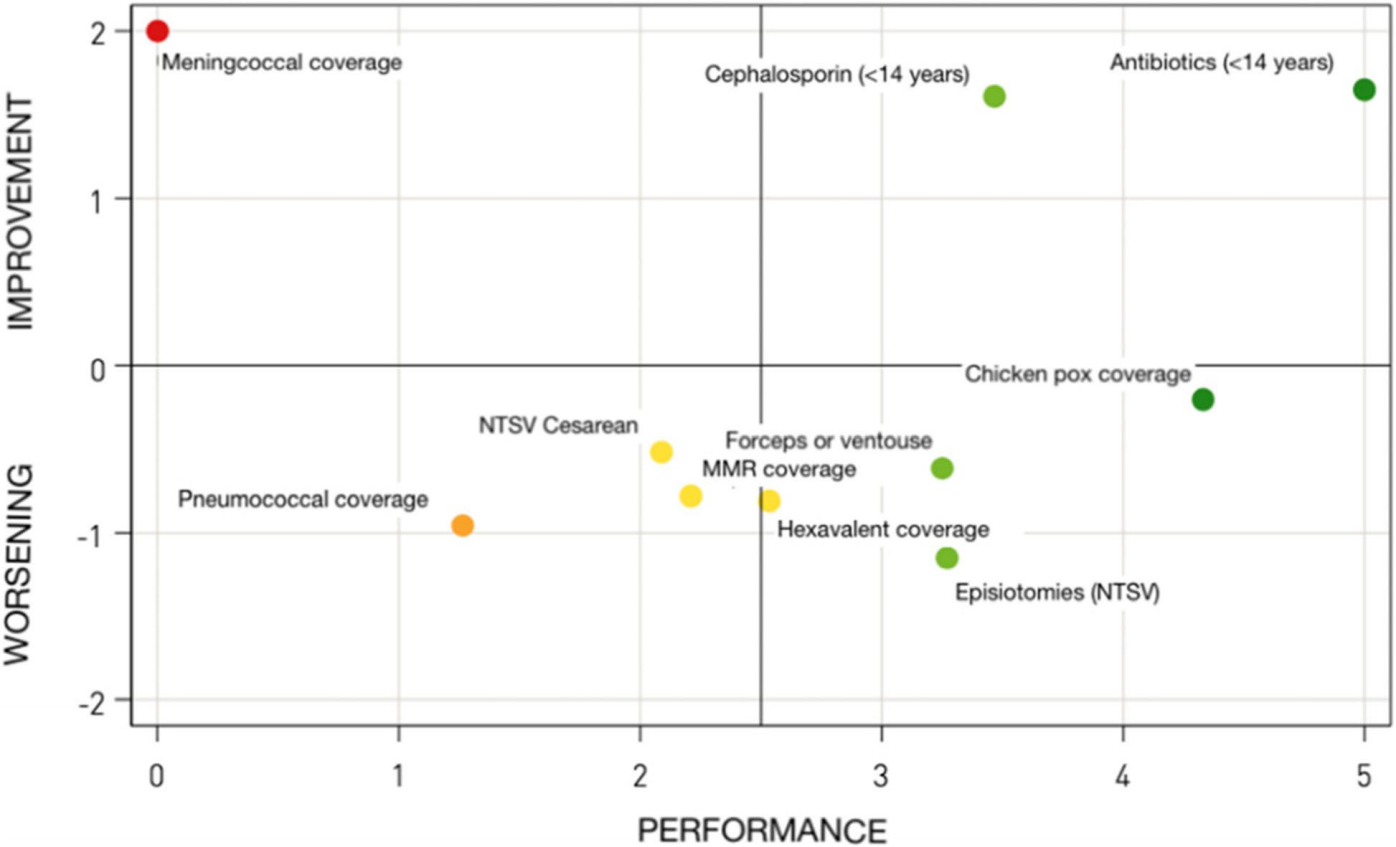
Regional performance map of maternal-child pathway

To conclude, the pentagrams are linked to performance maps (Fig. 5), to facilitate a reading of the dynamics of the regional health systems. Each of these includes all evaluation indicators of each pathway and reports, for each indicator, its performance in the current year and its variation with respect to the previous year. In the performance maps, the trend – y-axis – is calculated, for each selected indicator, as a percentage change 2020–2019 (rescaled to a -2 to + 2 range for all indicators). The performance – x axis – on the other hand, corresponds to the evaluation score attributed to each indicator in the year 2020. Thus, it becomes possible to identify four quadrants of reference: if the indicator is located in the upper right quadrant, it presents an excellent performance, both in terms of positioning with respect to the other regions, and in terms of capacity for improvement between 2019 and 2020. If the figure is in the upper left quadrant, it means that it has improved between 2019 and 2020 but that its 2020 performance level still cannot be considered satisfying. If the indicator is located in the lower right quadrant, it obtains a good assessment, i.e. it pursues a good result in 2020, but registers a worsening trend, which should alert its stakeholders. To conclude, if the figure is located in the lower left quadrant, its evaluation is lower than the other regions and with a worsening trend.

## The IRPES in times of Covid-19

The COVID-19 pandemic revolutionised not only the organisation within health care systems, but clearly affected performance evaluation systems too. Italian regions and MeS Lab immediately realised that the set of indicators normally adopted as the basis for monitoring the improvement process and the effort—intended to improve quality and reduce intra-regional and interregional unwarranted variation—certainly had a very different meaning in a pandemic period. Thus, in 2020, the IRPES has been called upon to integrate the classic “pre-Covid” monitoring metrics, which still has been carried on, with new information.

Starting from the second semester of 2020, the twelve regions adhering to the network of regions identified a preliminary set of 63 indicators to be monthly monitored (Table 2).

**Table 2:** Indicators of the Performance Evaluation System of Regional Health Systems for the year 2020

**Oncological treatments**
Surgical interventions for breast cancer (priority A)
Surgical interventions for prostate cancer (priority A)
Surgical interventions for colon cancer (priority A)
Surgical interventions for rectal cancer (priority A)
Surgical interventions for lung cancer (priority A)
Surgical interventions for uterine cancer (priority A)
Surgical interventions for melanoma (priority A)
Surgical interventions for thyroid cancer (priority A)
Patients treated with chemotherapy drugs
**Time sensitive clinical networks**
AMI STEMI hospitalizations
Ischemic stroke hospitalizations
**Cardio-circulatory area**
Angioplasty surgeries
Aorto-coronary by-pass surgeries
**Emergency department**
*Number of accesses in emergency departments*
Median length of stay in the emergency departments
**System indicators**
*Urgent hospitalizations*
*Elective hospitalizations*
*Surgeries for femoral neck fractures*
*Elective surgical hospitalizations*
***Outpatient care and Diagnostics***
*Volumes of outpatient services*
*Diagnostic imaging volumes*
***Ambulatory care – First visits***
*Volumes for first cardiology examination*
*Volumes for the first vascular surgery examination*
*Volumes for the first endocrinological examination*
*Volumes for the first neurological examination*
*Volumes for first eye examination*
*Volumes for first orthopaedic examination*
*Volumes for first gynaecological examination*
*Volumes for first otorhinolaryngological examination*
*Volumes for first urological examination*
*Volumes for first dermatological examination*
*Volumes for first physiatric examination*
*Volumes for the first gastroenterological examination*
*Volumes for the first oncological examination*
*Volumes for the first pneumological examination*
***Ambulatory care – Follow-ups***
Volumes for cardiology check-ups
Volumes for vascular surgery check-ups
Volumes for endocrinological check-ups
Volumes for neurological check-ups
Volumes for ophthalmological check-ups
Volumes for orthopaedic check-ups
Volumes for gynaecological check-ups
Volumes for otorhinolaryngological check-ups
Volume per urological examination
Volume per dermatological check-up
Volumes for physiatry check-ups
Volumes for gastroenterological check-ups
Volumes for oncological check-ups
Volumes per pulmonary check-up
***Mental Health***
*Mental Health—Volumes of home services*
*Mental health—Volumes of services provided in the territory*
**Home care**
Home care—Volumes for ADI/ADP home visits
***Exemptions***
*Exemptions—Volumes new exemptions for rare diseases*
*Exemptions—Volumes of new exemptions for chronic diseases*
*Exemptions—Volumes of new exemptions for disability*
*Exemptions—Volumes of new exemptions for income*
*Exemptions—Volumes of new exemptions for other conditions*
**Pharmaceuticals**
Consumption (in packs) of anti-diabetic drugs on the territory
Consumption (in packages) of substances that act on the renin-angiotensin system in the territory
*Consumption (in packages) of medicines for mental health in the territory*
**Oncological screenings**
Mammography screening volume trends
Cervical screening volume trend
Colorectal screening volume trend

The indicators were identified partly based on prior pioneering experiences [45], which primarily focused on volumes of activity—mostly inpatient and outpatient services. In order to properly identify important drops in healthcare service supply, computed by juxtaposing volumes of service provided in each month with those registered in 2019, the indicators were calculated with a three-month lag. Adding the concept of resilience to classic performance evaluation measures was therefore an innovative element of the collaboration by the MeS Lab with the network of regions. Indeed, not only on April 23^rd^ 2021^1^ the network of regions published outcome measures of the aforementioned 63 indicators for the year 2020, but on June 22^nd^, it publicly disclosed results of those 400 measures composing the traditional performance measurement system^2^ as well. Thus, while facing the first wave of the pandemic, it became clear to both the MeS Lab and network of regions that the evaluation system had shifted its focus also to a new area of investigation. In particular, to the extent to which the balance of priorities of the health systems have changed during 2020, the more classic dimensions of evaluation – i.e. sustainability, effectiveness, quality of the processes, appropriateness, equity, etc. – have been integrated with the dimension of resilience. This term is conceptually interpreted by the network of regions in a broad sense, as the ability of healthcare systems to respond to the COVID-19 crisis. Hence, resilience has been operationally defined as the ability of health systems to respond to the needs of the population, while containing the contraction in the volume of services provided. This represents a very pragmatic meaning of the broader concept of resilience, which has the merit of focusing the work of analysis and evaluation in a precise manner.

More specifically, the network of regions established—based on population’s needs—three kinds of indicators, so as to generate a timely study of its own resilience capacity:

1. Non-deferrable activities: services in the oncological and cardiovascular domains for which healthcare systems have been requested by legislation and national directives to make an attempt to keep volume contraction to a minimum during 2020 (indicators highlighted in pink, in Table 1). Among these, e.g., the aorto-coronary by-pass surgeries.
2. Quality of care: indicators that have been designed to assess the quality of services, e.g. evaluation of the proportion of femoral neck fractures operated within two days.
3. Overall response of the health system: these indicators are helpful on one hand to offering an overview of the overall situation; on the other, at highlighting—at the macro level—those areas in which specific regional systems are currently being called upon to undertake a revitalisation/rebound effort so as to regain the services that were not supplied during 2020. This category includes, e.g., the number of (missed/delayed) scheduled surgical hospitalisations.

**Fig. 6:**
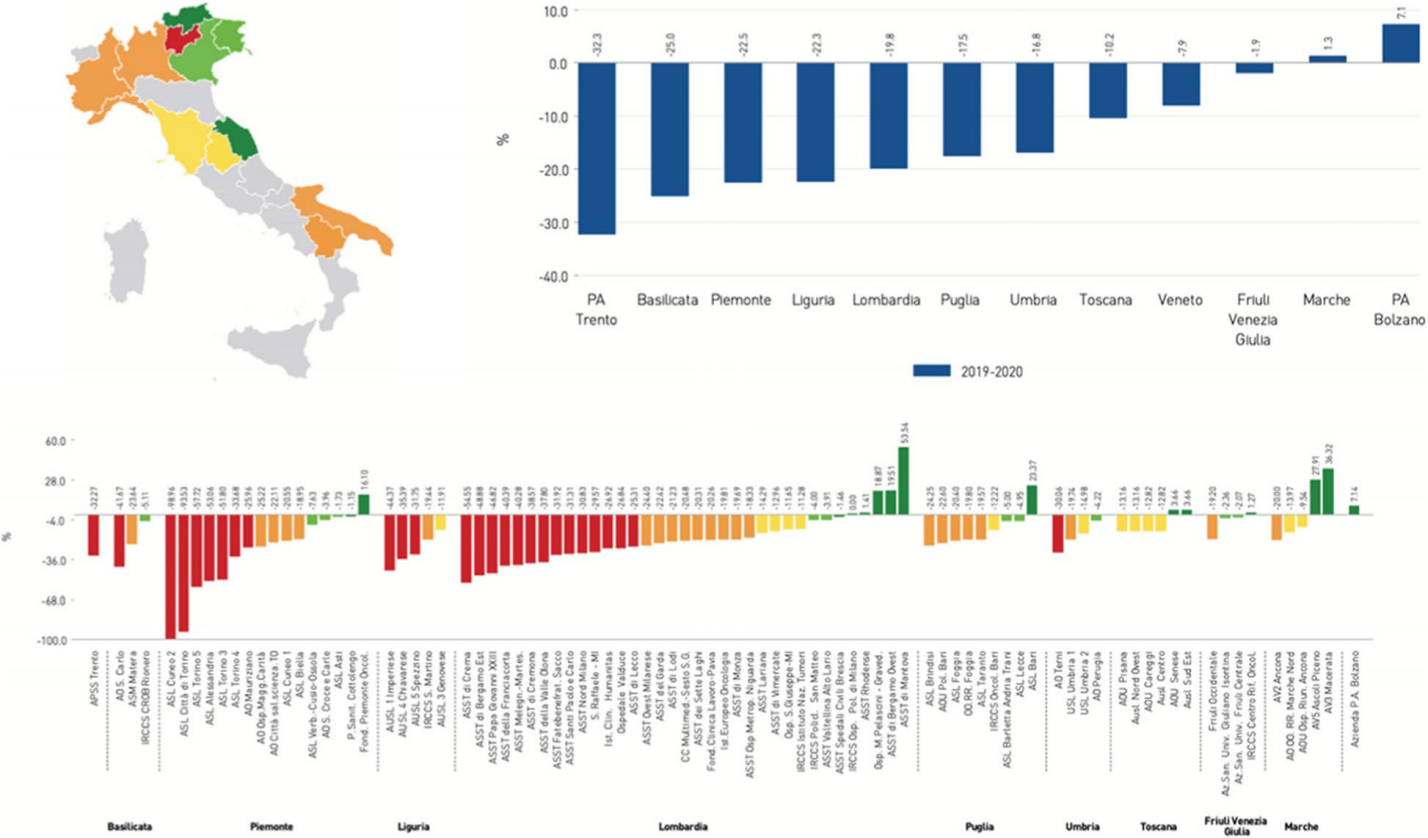
Set of resilience graphs for indicator “a”

**Fig. 7:**
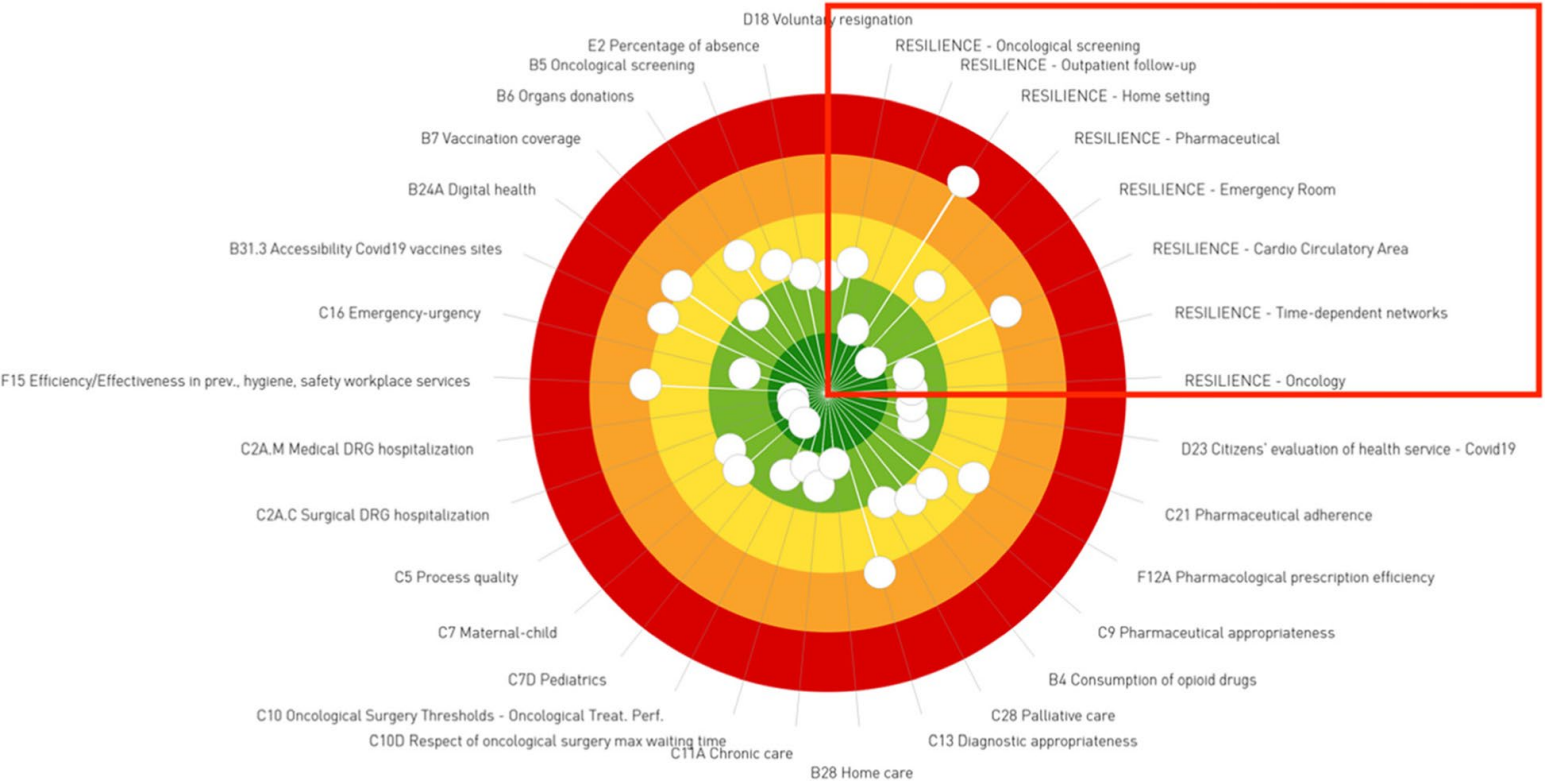
The “dartboard” of Region 3, year 2020. Resilience indicators are depicted in the upper right part of the dartboard (red rectangle)

Figure 6 illustrates an example of a set of resilience graphs, which maintain their classical design, but illustrate different measures: the two histograms represent the difference of volumes from 2020 to 2019 respectively for regions (blue) and their companies (five colours); the map summarises the performance of the regions. In terms of graphical representation, the resilience dimension has also been added to the classical dartboard designed by the MeS Lab, by occupying the upper right quadrant of the shape (Fig. 7). Considering Fig. 7, it is possible to conclude that “Region 3” has been considerably resilient in all fields of study, with the exception of home care.

**Fig. 8:**
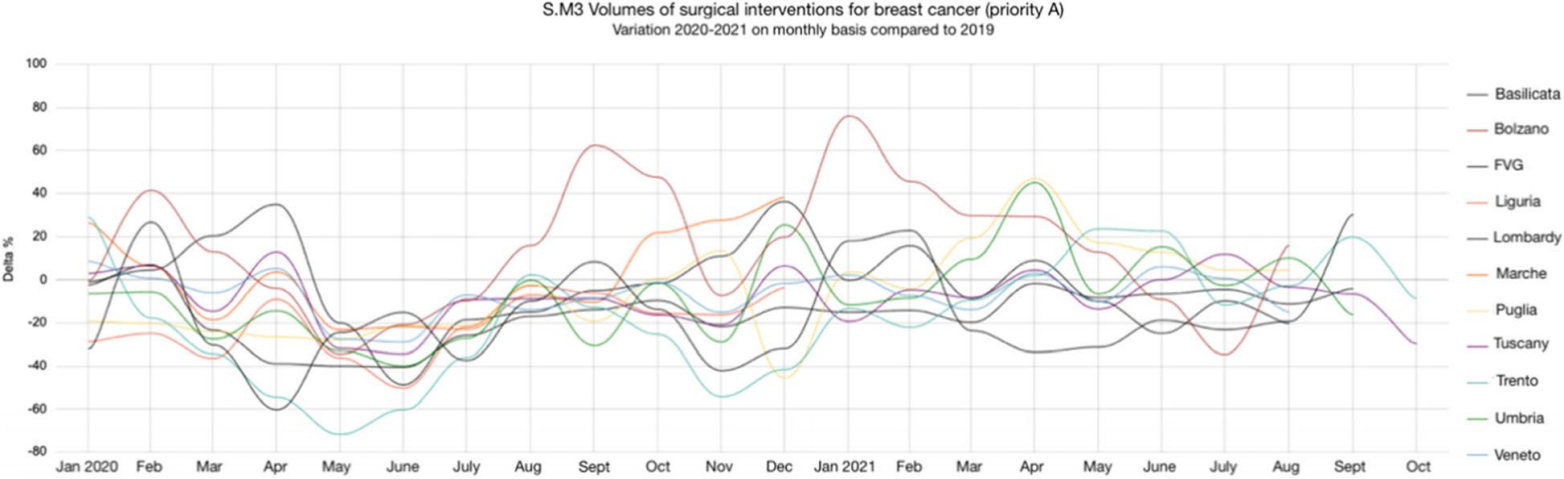
Monthly monitoring of Surgical interventions for breast cancer (priority A), from January 2020 until October 2021

Since 2020, Regions decided to pursue the monitoring of the identified resilience indicators, with the aim of having a continuously (monthly) up to date information on how healthcare providers were coping with non-deferrable activities, such as oncological care (Fig. 8). According to Fig. 8, all regions witnessed a decrease in surgical interventions for breast cancer during the first wave of the pandemic (March-June 2020). However, different performances can be observed from August 2020 onwards. Indeed, if some regions were able to recover the volumes of surgical interventions, others experienced greater difficulties in adjusting to the disruption.

In order to further improve the timeliness and relevance of the evaluation system – two key characteristics that the IRPES considered to be an added value with respect to the baseline tool during the pandemic emergency – three additional elements were investigated for the year 2020, which, when combined with the three groups of indicators mentioned above, provided a more wholesome monitoring of the performance of regions during the pandemic emergency compared to the evaluation tool adopted until then. The first one is a survey of the Italian population. This project aimed at grasping the perception of citizens – COVID-19-related patients as well as non-related ones – of the effectiveness of health services during the pandemic. It was conducted online between December 22, 2020, and January 28, 2021, with a total of 12,322 interviews. The second element was related to the assessment—in terms of completeness, comprehensibility and readability of the text—of the web pages of all 108 Italian Local Health Authorities adhering to the IRPES, which delivered information on the COVID-19 immunization. To conclude, the third element investigated for the year 2020 was related to the COVID-19 vaccination coverage in the various Italian regions. 18 indicators are currently weekly processed from data provided by the Civil Protection and the Ministry of health. For each indicator, three elements are depicted:

i. the regional value;
ii. the trend compared to the previous week;
iii. the scatter plot combining the two information: current situation and acceleration capacity in the past week.

**Fig. 9:**
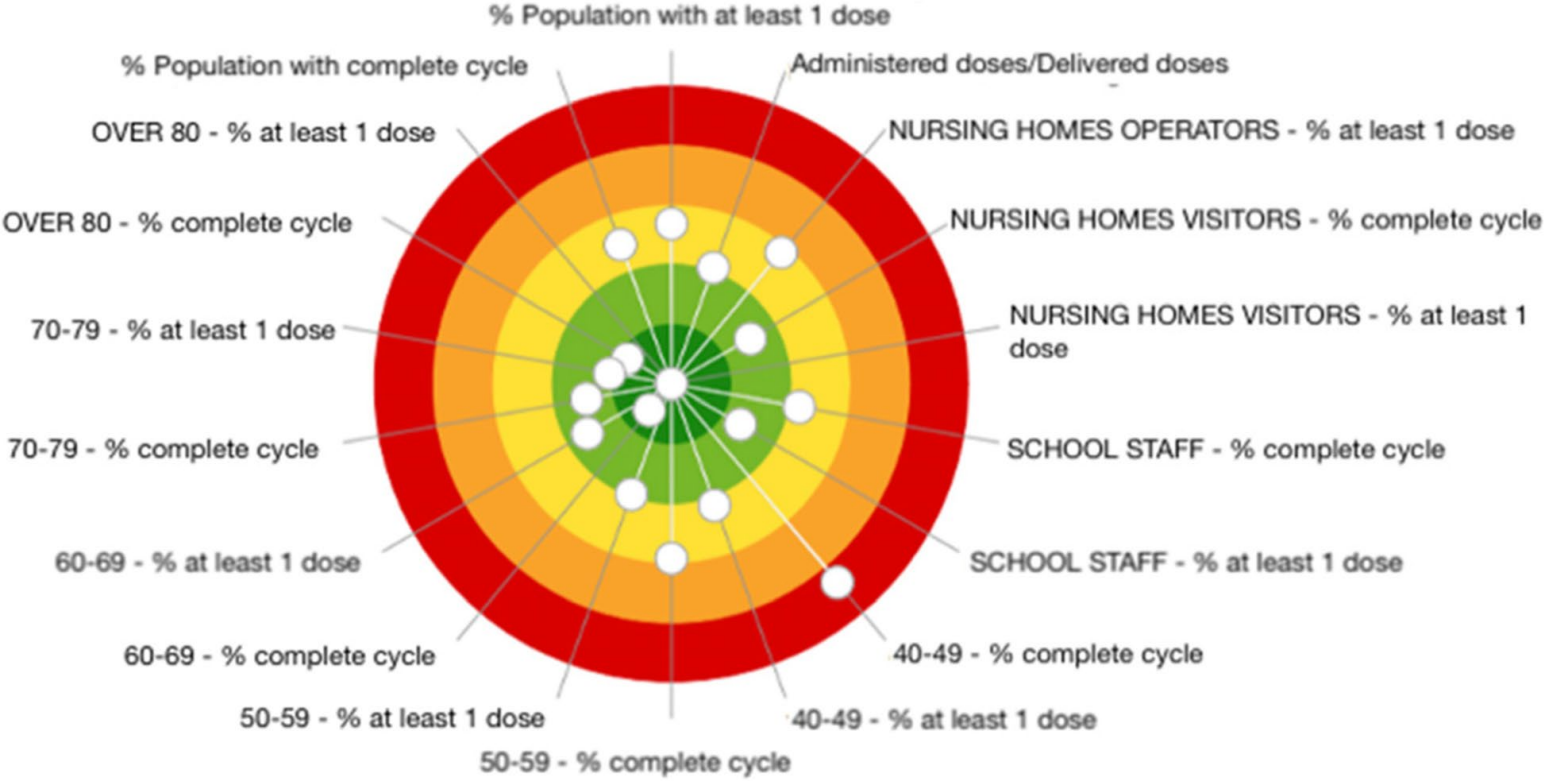
Example of a regional target, referred to COVID-19 Vaccination Plan Monitoring

Moreover, regional dartboards (Fig. 9) are constructed from the scores of the different indicators. Reports are updated weekly and publicly available at: https://performance.santannapisa.it/pes/start/vaccini.php.

## Conclusions

This paper intends to describe the performance evaluation system developed by a collaboration of Italian Regions, and promoted by MeS Lab. The manuscript highlights key characteristics of the IRPES and its evolution in front of the pandemic.

Nine main features were reported and described, namely voluntary adhesion, public disclosure and transparency, evidence based, systematic evaluation and benchmarking, shared design, multidimensionality, timeliness, dynamism, and investment in intuitive graphical representation.

Some of these represent “preconditions” for any HSPA. In order for performance assessment to be effective, it needs to reliable, informative and accountable: “evidence based”, “systematic benchmarking” and “public disclosure” are three characteristics that the IRPES share with PNE and NSG as an HSPA “least common multiple”. Remaining characteristics specifically relate to the goal the IRPES is aiming at: providing regional managers with a useful tool to steer regional health systems. This goal is IRPES-specific, it is not fully shared by PNE and NSG and this explains its uniqueness. Voluntary adhesion, shared design, multidimensionality, timeliness, dynamism, investment in intuitive graphical representation are six unique features of the IRPES that respond to its mission of being an effective managerial tool in the hands of regional administrators.

Responding to the pandemic did not require redefining the key characteristics of the system, but rather leveraging them in a different way. In particular, shared design and the dynamic approach allowed the system to be timely reframed, by developing previous experiences and offering a prompt monitoring system to regional managers. The rapid inclusion of resilience among the dimensions of the IRPES was the result of the agile and pragmatic approach the system intrinsically endorses.

Further details can be retrieved at https://performance.santannapisa.it/ or requested at network.regioni@santannapisa.it.

## Data Availability

Data mentioned in the manuscript are freely available on the Internet (www.performance.santannapisa.it).

## Footnotes

1 https://youtu.be/ywKWtXFFP1I

2 https://youtu.be/jlxXpUAjWTQ
